# Serum soluble interleukin‐2 receptor levels in hairy cell leukaemia as a marker of tumour burden with prognostic value and as a tool for disease monitoring

**DOI:** 10.1111/bjh.70059

**Published:** 2025-07-29

**Authors:** Francesco Angotzi, Alessandro Cellini, Nicolò Danesin, Simone Zoletto, Andrea Serafin, Chiara Adele Cavarretta, Arianna Bevilacqua, Laura Forlani, Alessia Tonini, Federica Frezzato, Laura Bonaldi, Marco Pizzi, Diego Faggian, Livio Trentin, Andrea Visentin

**Affiliations:** ^1^ Hematology Unit, Department of Medicine University of Padova Padova Italy; ^2^ Immunology and Molecular Oncology Unit Veneto Institute of Oncology (IOV‐IRCCS) Padova Italy; ^3^ Department of Medicine, Surgical Pathology and Cytopathology Unit University of Padova Padua Italy; ^4^ Department of Integrated Diagnostic Medicine, Laboratory Medicine Unit University of Padova Padova Italy

**Keywords:** hairy cell leukaemia, interleukins, purine analogues, residual disease

## Abstract

Leukaemic cells from hairy cell leukaemia (HCL) secrete a soluble form of the interleukin‐2 receptor (sIL‐2R) which is measurable in serum. Previous evidence suggested that sIL‐2R may correlate well with tumour burden and demonstrated a reduction in sIL‐2R levels after therapy with recombinant interferon‐α2. We evaluated the role of sIL‐2R as a new prognostic factor and as a tool for disease monitoring. sIL‐2R correlated well with other markers of neoplastic bulk and markedly decreased after treatment, with lower levels achieved in patients reaching complete remission (*p* = 0.002) or negative minimal residual disease (MRD, *p* = 0.034). Post‐treatment levels ≤827 kU/L were strongly predictive of longer time to next treatment (TTNT) (median NR vs. 4.87 years; hazard ratio [HR]: 0.10; 95% confidence interval [CI]: 0.02–0.24; *p* < 0.001) and higher 5‐ and 10‐year TTNT rates (5‐year: 93% vs. 45%; 10‐year: 83% vs. 11%). Furthermore, a ≥50% increase in sIL‐2R levels over any 1‐year interval during follow‐up predicted impending relapse. In a context where patients with HCL are expected to achieve a life expectancy comparable to that of the general population, sIL‐2R has the potential to serve as a non‐invasive tool alongside MRD to predict relapse and to identify those patients who may fail to derive the most benefit from current treatments.

## Graphical abstract

Soluble interleukin‐2 receptor (sIL‐2R) is readily measurable in the serum of individuals affected by hairy cell leukaemia (HCL) and has the potential to become a marker of clinical utility. sIL‐2R levels reflect total HCL disease burden as demonstrated by their correlation with other indicators of tumour load. Post‐therapy sIL‐2R levels retain prognostic potential and are able to identify patients at early risk of relapse. Moreover, during yearly disease monitoring, an increase ≥50% in sIL‐2R levels over a 1‐year interval is predictive of impending relapse.

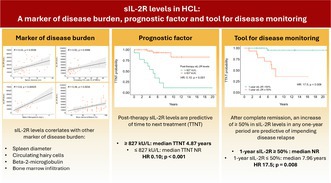

## INTRODUCTION

The interleukin‐2 receptor (IL‐2R) is expressed by various cell types and plays a pivotal role in immune regulation.^1^ A soluble form (sIL‐2R) is secreted in the serum during infections, inflammatory, autoimmune and neoplastic conditions, including hairy cell leukaemia (HCL).^2^, ^3^ Elevated serum sIL‐2R levels in HCL were initially demonstrated in two seminal works, which also noted decreasing levels after treatment with recombinant interferon‐α2 (rIFNα‐2).^4^, ^5^ These results were then confirmed in patients treated with rIFNα‐2 or pentostatin (DCF), showing good correlation between sIL‐2R levels and tumour burden.^6^, ^7^, ^8^, ^9^, ^10^, ^11^, ^12^, ^13^, ^14^, ^15^

While these studies provided a theoretical basis for the use of sIL‐2R as a disease marker, a more concrete use of sIL‐2R in clinical practice remains unexplored. Indeed, only one study reported elevated sIL‐2R levels several years before disease relapse in eight patients,^16^ and another assessed sIL‐2R for guiding rIFNα‐2 tapering during maintenance therapy.^17^ Moreover, only a few reports demonstrated reduction in sIL‐2R levels after therapy with the most commonly used purine analogue cladribine (2CDA).^18^, ^19^, ^20^, ^21^

We hypothesized that sIL‐2R could represent a non‐invasive and versatile marker in HCL. In this study, we aimed to complement and expand the aforementioned evidence by reporting our experience with the use of sIL‐2R serum levels in HCL, which are commonly measured at our institution.

## METHODS

We designed a retrospective cohort study enrolling patients with classical HCL diagnosed and treated at the haematology unit of Padua university hospital from 1992 until 2022. Eligible patients needed a histologically confirmed diagnosis of classical HCL, documented BRAF‐V600E mutation in the bone marrow (via allele‐specific polymerase chain reaction (PCR)) and either sIL‐2R levels measured before or after any line of therapy, or two or more consecutive sIL‐2R measurements taken at 1‐year intervals during disease monitoring after therapy. The latter formed a disease monitoring cohort used to evaluate sIL‐2R levels during follow‐up. Response was assessed at 6 months post‐treatment with purine analogues and according to the 2017 consensus guidelines.^22^

The study received ethics committee approval (number: 4218/A0/17) and was conducted in accordance with the declaration of Helsinki. All patients provided written informed consent.

We collected individual patient data from medical records, including laboratory values, CD38 expression evaluated by flow cytometry, the presence of splenomegaly evaluated by clinical examination, spleen dimensions measured by CT scan or abdominal ultrasound and the percentage of bone marrow infiltration by HCL cells recorded before treatment alongside sIL‐2R levels. Data regarding therapy, response, minimal residual disease (MRD) evaluated by immunohistochemistry (IHC) according to the consensus guidelines with antibodies against CD20, DBA44 and/or BRAF‐V600E^22^ and sIL‐2R levels at the time of response evaluation and annually post‐treatment were also collected. sIL‐2R levels were measured in patients' serum by ELISA (adult reference values 250–730 kU/L; Siemens IMMULITE® platform, Germany).

To evaluate the relationship between response depth and sIL‐2R, we assessed the correlation between clinical response, namely complete remission (CR), partial remission (PR) and stable disease (SD) and post‐therapy sIL‐2R levels. The same was done with MRD status.

### Statistical analysis

Overall survival (OS) was defined from diagnosis until death or last visit (censored), and time to next treatment (TTNT) from response assessment after the last line of therapy until the subsequent treatment. Continuous variables were summarized as median and interquartile range (IQR); categorical variables as counts and percentages. Mann–Whitney *U*‐test and chi‐square/Fisher's exact tests were used for group comparisons. The correlation between sIL‐2R levels and other variables was explored by logistic regression (categorical variables) and Spearman's rank (continuous variables). In order to further explore the association of post‐therapy sIL‐2R levels with response depth, we performed univariate and multivariate logistic regressions for patients achieving a CR versus PR/SD. Survival curves were estimated through the Kaplan–Meier method and compared with log‐rank tests. The prognostic potential of pre‐therapy and post‐therapy sIL‐2R levels was assessed by evaluating the association of log‐transformed sIL‐2R levels as a continuous variable with TTNT and OS. Log‐transformation of sIL‐2R levels was used to enhance the interpretability of odds ratios (OR) and hazard ratios (HR). Univariate and multivariate analyses for time‐to‐event outcomes were performed by the Cox proportional‐hazards model and stratified log‐rank test. Statistically significant variables in univariate analyses were included in multivariate models. To identify an exploratory threshold of post‐therapy sIL‐2R levels to predict TTNT, we employed a maximally selected rank statistics analysis.^23^ Alongside the identified threshold, the prognostic power of both achieving post‐therapy levels ≤ to the test's upper limit of normal (ULN) and the magnitude of logarithmic reduction between pre‐ and post‐therapy levels was also evaluated.

Predictive performance was further evaluated by time‐dependent receiver operating characteristic (ROC) curve analysis.^24^, ^25^ The analysis was conducted according to the inverse probability of censoring weighting (IPCW) estimation of cumulative/dynamic (C/D) time‐dependent ROC.^26^, ^27^ Significance was set at *p* < 0.05. All analyses were performed with R software (version 4.3.1).

## RESULTS

### Patients' characteristics and their correlation with sIL‐2R

A total of 74 patients were included. Median age was 59.6 years (IQR: 20.4), and 61 patients (82.4%) were male. The presence of the BRAF‐V600E mutation was confirmed at the time of diagnosis for those diagnosed after 2013 (*n* = 59; 79.7%), while for those diagnosed before 2013, it was confirmed at a later time point either upon relapse (*n* = 4; 5.4%) or on frozen bone marrow samples (*n* = 11; 14.9%). Fifty‐six (75.7%) patients had both pre‐ and post‐therapy sIL‐2R measurements, 4 (5.4%) only post‐therapy, 5 (6.8%) only pre‐therapy levels and 9 (12.2%) only yearly sIL‐2R measurements after therapy and were only included in the disease monitoring cohort. This latter cohort was comprised of 54 (73%) patients in total. Baseline population characteristics are summarized in Table S1; there were no statistically significant differences in terms of baseline characteristics between the nine patients included only in the monitoring cohort and the rest (data not shown).

**FIGURE 1:**
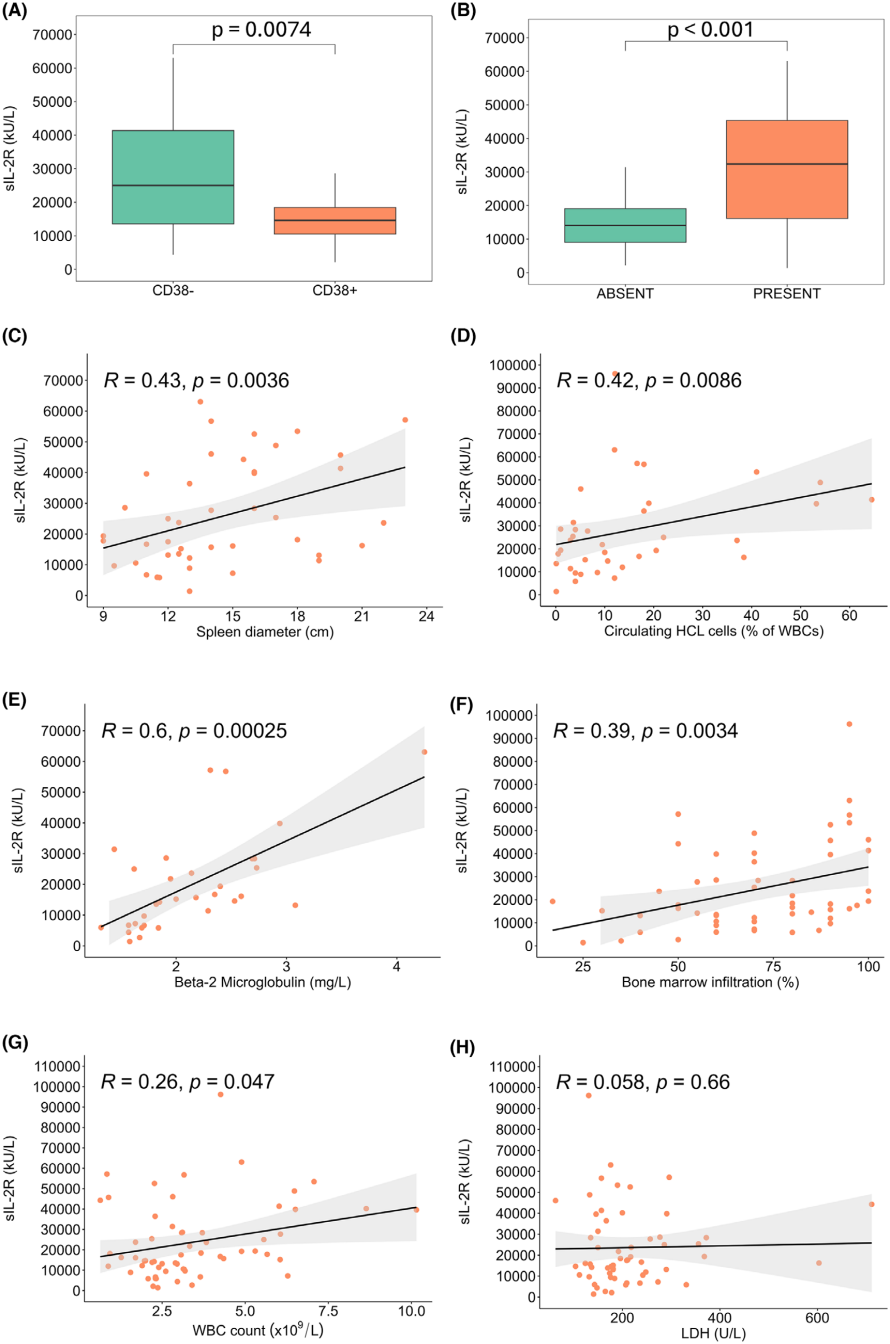
Correlation of sIL‐2R levels with other markers of disease burden. (A, B) Boxplots showing correlation between median sIL‐2R levels and CD38 expression status (A) and with the presence/absence of splenomegaly (B); (C) correlation with spleen diameters; (D) correlation with the % of circulating hairy cells out of all WBCs; (E) correlation with beta‐2‐microglobulin levels; (F) correlation with the % of bone marrow infiltration; (G) correlation with WBC count; (H) correlation with LDH levels. LDH, lactate dehydrogenase; sIL‐2R, soluble form of the interleukin‐2 receptor; WBC, white blood cell count.

The median pre‐therapy sIL‐2R level was 17 510 kU/L (IQR: 20040 kU/L). sIL‐2R levels were lower in CD38+ patients (median 14 570 vs. 25 190 kU/L, *p* = 0.0074) (Figure 1A), higher in those with splenomegaly (median 28 350 vs. 14 060 kU/L, *p* = 0.0008) (Figure 1B) and correlated with both the presence of splenomegaly (OR: 3.06; *p* = 0.006) and spleen diameters (*R* = 0.43; *p* = 0.0036) (Figure 1C). Serum sIL‐2R levels showed a positive correlation with the percentage and number of circulating hairy cells (*R* = 0.41, *p* = 0.009 and *R* = 0.42; *p* = 0.0086), β2‐microglobulin (*R* = 0.61; *p* < 0.001), the degree of bone marrow infiltration (*R* = 0.39; *p* = 0.0034) and WBC count (*R* = 0.26; *p* = 0.047) (Figure 1D–G), but not with lactate dehydrogenase (LDH) levels (*R* = 0.06; *p* = 0.66) (Figure 1H).

### Correlation of sIL‐2R levels and response to treatment

All of the patients with available post‐therapy sIL‐2R values (*n* = 60) were treated with purine analogues, 52 (86.7%) with 2CDA and 8 (13.3%) with DCF. Fifty‐six (93.3%) had both pre‐ and post‐therapy sIL‐2R levels available. In this group, the median pre‐therapy sIL‐2R level was 16 475 kU/L (IQR: 17838) and decreased to 599 kU/L (IQR: 388.75) after therapy (*p* < 0.001; median reduction: 15946.5 kU/L, IQR: 18355) (Figure S1). An exploratory analysis was conducted on six patients treated with R‐Vemurafenib upon relapse, yielding similar results (Figure S2).

**FIGURE 2:**
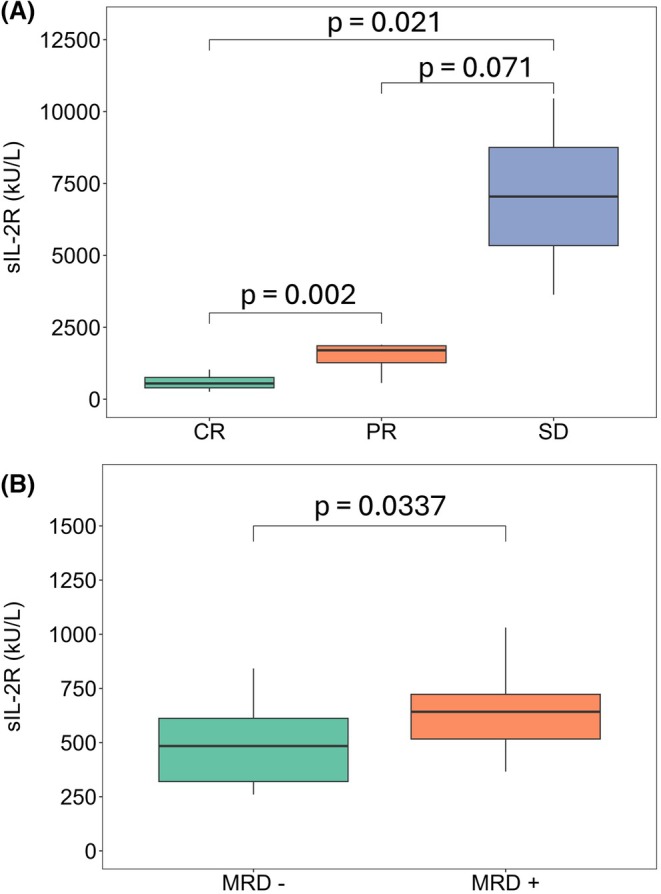
Boxplots comparing post‐therapy sIL‐2R levels according to response type (A) and MRD status (B). sIL‐2R, soluble form of the interleukin‐2 receptor.

Of 60 patients, 52 (86.7%) achieved a CR, 6 (10%) achieved a PR and 2 (3.3.%) achieved SD. Patients achieving a CR had a median post‐therapy sIL‐2R level of 546 kU/L (IQR: 360 kU/L), which was significantly lower than those achieving PR (1700 kU/L, IQR: 589 kU/L; *p* = 0.002) or SD (7047 kU/L, IQR: 3413 kU/L; *p* = 0.021) while there was no significant difference between patients achieving PR versus SD (*p* = 0.071) (Figure 2A). In univariate analysis, the percentage of circulating hairy cells was a significant predictor of response depth along with post‐therapy sIL‐2R; only the latter remained significant after multivariate analysis (Tables S2 and S3). MRD+ patients had significantly higher median sIL‐2R levels compared with MRD‐ ones (642 kU/L vs. 484 kU/L, *p* = 0.034; Figure 2B).

### Prognostic and predictive impact of sIL‐2R

**FIGURE 3:**
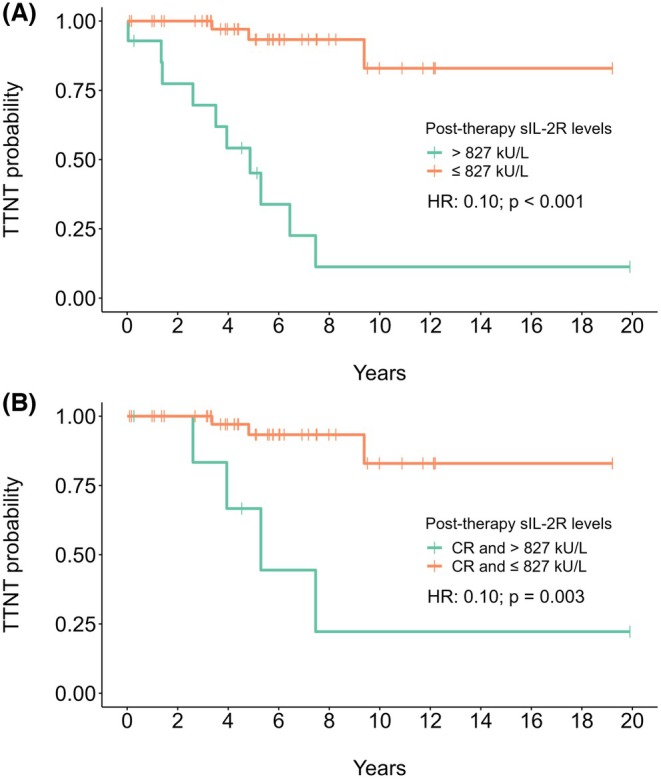
Survival curves with predicted TTNT based on measured post‐therapy sIL‐2R levels at the time of response evaluation for the 827 kU/L cut‐off. In the whole cohort (A) and only in patients concomitantly achieving CR (B). CR, complete remission; sIL‐2R, soluble form of the interleukin‐2 receptor; TTNT, time to next treatment.

Post‐treatment sIL‐2R levels, treated as a continuous variable and log‐transformed, correlated with TTNT (HR: 15.6; 95% confidence interval [CI]: 4.9–50.0; *p* < 0.001), but not with OS (HR 1.4; 95% CI: 0.4–5.1; *p* = 0.63) in univariate analysis. Age was the only variable correlated with OS (HR: 1.13; 95% CI: 1.03–1.26; *p* = 0.012; Table 1 and Table S4). Also, the association of post‐therapy sIL‐2R levels with TTNT remained significant irrespective of the treatment era (pre‐2015 vs. post‐2015; *p* for interaction = 0.28) (Table 1). Along with disease status, post‐therapy sIL‐2R levels remained a significant predictor of TTNT in multivariate analysis (Table 2). Furthermore, the addition of post‐therapy sIL‐2R levels to a base prognostic model including only disease status and response significantly improved model performance (likelihood‐ratio test, *p* = 0.0006). Through maximally selected rank statistics, the value of 827 kU/L was identified as the best cut‐off point to predict TTNT (Figure S3). Achieving post‐therapy sIL‐2R levels ≤827 kU/L was significantly associated with longer TTNT (median NR vs. 4.87 years; HR: 0.10; 95% CI: 0.02–0.24; *p* < 0.001) and higher 5‐year and 10‐year TTNT rates compared to patients with sIL‐2R levels >827 kU/L (5‐year TTNT: 93% vs. 45%; 10‐year TTNT: 83% vs. 11%) (Figure 3A). Among patients with sIL‐2R levels >827 kU/L, 4/14 (28.6%) patients had relapsed/refractory disease, against 3/46 (6.5%) in the ≤827 kU/L group (*p* = 0.075). The 827 kU/L cut‐off remained significant in the newly diagnosed group (HR: 0.06; 95% CI: 0.01–0.30; *p* < 0.001) (Figure S4), and also while adjusting for disease status (newly diagnosed vs. relapsed/refractory; stratified log‐rank test *p* < 0.001) in the whole cohort. Since the predicted value was very close to the test's original ULN (730 kU/L), we also explored the predictive potential of achieving post‐therapy sIL‐2R levels within this ULN in a sensitivity analysis. Again, achieving post‐therapy sIL‐2R values ≤ULN was associated with longer TTNT (median NR vs. 6.44 years; HR: 0.16; 95% CI: 0.04–0.58; *p* = 0.005) and higher 5‐year and 10‐year TTNT rates compared with those who did not (5‐year TTNT 92% vs. 63%; 10‐year TTNT 79% vs. 39%; Figure S5). When restricting the analysis to patients in CR, the 827 kU/L cut‐off was again able to predict longer TTNT (median NR vs. 5.29 years; HR: 0.10; 95% CI: 0.02–0.47; *p* = 0.003) (Figure 3B). A trend towards shorter TTNT with higher post‐therapy sIL‐2R levels was observed among both MRD+ (HR: 3.5; 95% CI: 0.46–25.9; *p* = 0.23) and MRD‐ patients (HR: 9.82; 95% CI: 0.07–129; *p* = 0.36). The degree of reduction in sIL‐2R after therapy also correlated with TTNT (HR: 0.30; 95% CI: 0.15–0.60; *p* < 0.001), and a cut‐off of ≥3‐log reduction was predictive of longer TTNT (median NR vs. 7.46 years; HR: 0.19; 95% CI: 0.05–0.69; *p* = 0.011) (Figure S6).

**TABLE 1:** Univariate analysis for TTNT.

	HR	95% CI	*p*‐value
Post‐therapy log(sIL‐2R)	15.6	4.9–50.0	**<0.001**
Pre‐therapy log(sIL‐2R)	1.21	0.61–2.43	0.58
Age	0.99	0.9–1.0	0.55
CD38+	1.27	0.40–4.0	0.68
WBC count	1.03	0.76–1.40	0.84
Splenomegaly	0.36	0.48–4.27	0.52
Spleen diameter	1.06	0.92–1.23	0.41
LDH	0.99	0.99–1.0	0.68
B2M	0.44	0.07–2.78	0.39
% of bone marrow HCs before therapy	0.99	0.97–1.02	0.81
MRD+	1.94	0.32–11.9	0.47
Treatment era (pre‐2015 vs. post‐2015)	1.55	0.46–5.10	0.50
Post‐therapy log(sIL‐2R) × Treatment era	0.28	0.03–ND	0.28
Disease status (ND vs. R/R)	0.15	0.05–0.48	**0.002**
Response depth (CR vs. PR)	0.11	0.03–0.43	**0.001**

*Note*: Statistically significant *p*‐values indicated in bold.

Abbreviations: B2M, beta‐2 microglobulin; CI, confidence interval; CR, complete remission; HCs, hairy cells; HR, hazard ratio; LDH, lactate dehydrogenase; MRD, minimal residual disease; ND, newly diagnosed; ND, not determinable; PR, partial remission; R/R, relapsed/refractory; sIL‐2R, soluble form of the interleukin‐2 receptor; TTNT, time to next treatment; WBC, white blood cell count.

**TABLE 2:** Multivariate analysis for TTNT.

	HR	95% CI	*p*‐value
Post‐therapy log(sIL‐2R)	13.01	2.68–63.1	**0.001**
Disease status (ND vs. R/R)	0.12	0.03–0.59	**0.009**
Response depth (CR vs. PR)	0.39	0.07–2.17	0.28

*Note*: Statistically significant *p*‐values indicated in bold.

Abbreviations: CR, complete remission; HR, hazard ratio; ND, newly diagnosed; PR, partial remission; R/R, relapsed/refractory; sIL‐2R, soluble form of the interleukin‐2 receptor; TTNT, time to next treatment.

To further enquire about the predictive value of post‐therapy sIL‐2R levels, we performed a time‐dependent ROC curve analysis that revealed high area under the curve (AUC) values for predicting TTNT in the first 3 years after therapy (AUC 1.00 for first and second year and 0.95 for third). While relatively high values of AUC >0.80 were observed up until the tenth year after therapy, these were once again particularly elevated 8 and 9 years after therapy (AUC for both 0.91) (Table 3). The cut‐off of 730 kU/L showed relatively high levels of sensitivity and negative predictive value (NPV) up until the 9th year after therapy, but comparatively lower specificity and positive predictive value (PPV; Table S5). However, other cut‐off points may be more informative at different time points. Indeed, the values of 842 and 827 kU/L were the ones with the highest Youden's indexes for the time points at which sIL‐2R performed better in terms of TTNT prediction (i.e. 3rd–4th year and 8th–9th) (Table S6). The latter value was also the one which showed the greatest discriminating potential in terms of TTNT after maximally selected rank statistics analysis, and thus, we further analysed its performance as a cut‐off at the same time points, yielding good sensitivity and specificity especially up to the third year after therapy (sensitivity: 100%; specificity: 80.9%) and at the eighth and ninth year (sensitivity: 84.6% for both years; specificity: 90.9% at year 8 and 90% at year 9) (Table 3).

**TABLE 3:** Results of time‐dependent ROC curve analysis on the predictive performance of post‐therapy sIL‐2R levels on TTNT displaying the AUC at different time points. Sensitivity, specificity, PPV, NPV and Youden's index for the 827 kU/L cut‐off at the same time points are also displayed.

Time in years	sIL‐2R	827 kU/L cut‐off				
	AUC	Sensitivity	Specificity	PPV	NPV	Youden's index
*t* = 1	1.00	100%	78.2%	7.2%	100%	**0.78**
*t* = 2	1.00	100%	80.0%	22.0%	100%	**0.80**
*t* = 3	0.955	100%	80.9%	28.9%	100%	**0.81**
*t* = 4	0.914	84.4%	81.1%	41.7%	97.0%	0.65
*t* = 5	0.862	74.7%	83.3%	51.6%	93.3%	0.58
*t* = 6	0.845	78.3%	85.7%	61.1%	93.2%	0.64
*t* = 7	0.882	81.8%	87.5%	70.4%	93.0%	0.69
*t* = 8	0.906	84.6%	90.9%	81.1%	92.8%	**0.76**
*t* = 9	0.912	84.6%	90.0%	79.6%	92.7%	**0.75**
*t* = 10	0.859	69.5%	85.7%	75.2%	81.9%	0.55

*Note*: Youden's index values >0.70 are indicated in bold.

Abbreviations: AUC, area under the curve; NPV, negative predictive value; PPV, positive predictive value; ROC, receiver operating characteristic; TTNT, time to next treatment.

**FIGURE 4:**
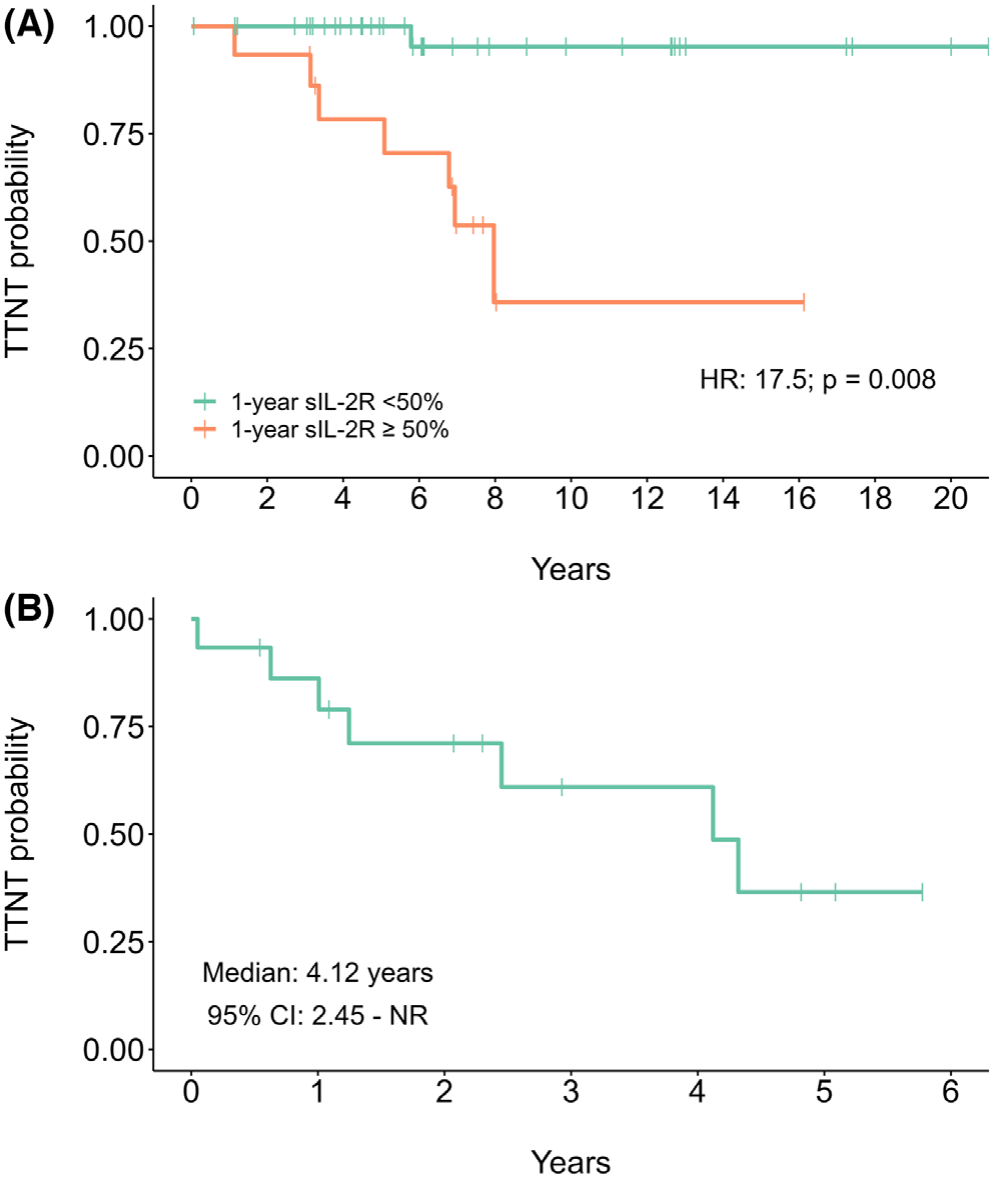
Survival curves showing predicted TTNT depending on the presence of a ≥50% increase in sIL‐2R levels during follow‐up (A) and predicted TTNT calculated from the time of the same increase in a landmark analysis (B). sIL‐2R, soluble form of the interleukin‐2 receptor; TTNT, time to next treatment. sIL‐2R, soluble form of the interleukin‐2 receptor; TTNT, time to next treatment.

In the disease monitoring cohort, the median follow‐up from the date of the first sIL‐2R measurement was 6.9 years (IQR: 4.5–11.4 years). Patients who experienced a ≥50% increase in sIL‐2R levels over any 1‐year interval displayed significantly shorter TTNT (median NR vs. 7.96 years; HR: 17.5; 95% CI: 2.14–143.4; *p* = 0.008) (Figure 4A), the median time from a ≥50% increase until the next line of treatment was 4.12 years (95% CI: 2.45–NR) (Figure 4B).

## DISCUSSION

sIL‐2R proved to be a reliable marker of disease burden, showing significant correlation with most indicators of HCL tumour load. While the WBC count is generally not elevated in HCL due to leucopenia and scarce circulating hairy cells,^8^, ^28^ sIL‐2R still correlated with peripheral blood hairy cell counts, possibly due to direct secretion of sIL‐2R in the bloodstream. In contrast, LDH did not correlate with sIL‐2R, suggesting how it may only reflect increased cell turnover rather than tumour burden. The feasibility of sIL‐2R as a marker of neoplastic bulk is further confirmed by its sharp post‐therapy decline and by its association with response depth. Collectively, these results corroborate and expand prior evidence on sIL‐2R as a marker of tumour burden in patients treated with rIFNα‐2 or DCF.^6^, ^7^, ^8^, ^10^, ^11^, ^12^, ^13^, ^14^, ^15^

While splenomegaly, CD38 expression, LDH, WBC count, circulating hairy cells and β‐2 microglobulin have been associated with worse outcomes in different studies,^29^, ^30^ we found no significant association between them and TTNT. In contrast, post‐therapy sIL‐2R levels were a robust predictor of TTNT in both univariate and multivariate analyses, with their prognostic value independent of pre‐therapy levels and instead tied to the degree of reduction or to absolute post‐therapy levels reflecting residual disease. Consistent with the effectiveness of purine analogues and the near‐normal life expectancy they confer,^28^, ^31^, ^32^, ^33^ sIL‐2R levels did not correlate with OS, and age was the only variable associated with OS.

While patients achieving MRD negativity had significantly lower sIL‐2R levels, post‐therapy sIL‐2R did not improve the prognostic performance of MRD regarding TTNT. This is likely due to only a limited part of our patients having MRD data (*n* = 36) and relatively short follow‐up (median 5.56 years; IQR: 3.18–10.9) which may have hindered MRD significance, as seen in other experiences with similar follow‐up.^34^ Nonetheless, complementing MRD with sIL‐2R remains promising, as suggested by 2017 consensus guidelines on HCL,^22^ given sIL‐2R's ease of measurement and low cost. Further research should explore combining sIL‐2R levels with more sensitive MRD assays such as MFC,^35^, ^36^ PCR^37^, ^38^, ^39^, ^40^, ^41^ and droplet digital PCR,^42^, ^43^ which outperform IHC.^44^

Using either 827 kU/L or the conventional ULN as a cut‐off, post‐therapy sIL‐2R effectively identified patients at higher risk of early relapse regardless of response depth. Indeed, those with levels >827 kU/L had a median TTNT of 4.87 years, shorter than the >10‐year progression‐free survival reported in patients treated with purine analogues.^45^, ^46^, ^47^ While the 827 kU/L cut‐off requires validation, especially in the relapsed/refractory setting given the low number of patients with previously treated HCL in our cohort (*n* = 7), it outperformed the ULN in terms of sensitivity and specificity (Table S5 and Table 4). sIL‐2R also proved useful in follow‐up: a ≥50% increase likely reflects active disease and impending relapse, identifying patients who may benefit from closer monitoring or early retreatment. However, since the ≥50% threshold is exploratory and was selected based on clinical experience, practicality (i.e. to minimize noise from minor fluctuations) and biological plausibility (i.e. a value that could represent an actual increase in tumour burden), it warrants validation in prospective cohorts. Serial monitoring of sIL‐2R could also be implemented in prospective trials investigating early retreatment before overt relapse. Notably, rituximab can eradicate residual disease after 2CDA treatment and has been used in MRD‐directed treatment strategies.^38^, ^48^

Limitations of this study include bias induced by its retrospective nature, and the lack of data regarding IGHV mutational status which has emerged as a viable prognostic factor, and like sIL‐2R, is associated with the presence of leucocytosis and splenomegaly.^49^ Furthermore, as stated previously, these results need confirmation in an independent cohort, especially regarding post‐therapy sIL‐2R cut‐off selection. Also, the lack of patients treated with R‐Vemurafenib or R‐2CDA in our cohort limits the generalizability of these results to these groups, which is relevant given the ability of rituximab to induce more profound responses,^28^, ^35^, ^48^, ^50^, ^51^ and the efficacy of R‐Vemurafenib in the relapsed/refractory setting.^37^

In conclusion, since HCL patients treated with purine analogues are expected to have a life expectancy comparable to that of the general population, tools to readily isolate patients who fail to fully benefit from treatment are especially useful. Pending validation, sIL‐2R has the potential to fulfil this role alongside MRD and to become a strong prognostic factor in HCL that closely correlates with total disease burden. Given its relative simplicity, low cost and non‐invasive nature, sIL‐2R may also act as a viable substitute to MRD evaluation in lower resource settings, for patients not keen to proceed to bone marrow evaluation after therapy or in the case of haemodilution of bone marrow samples which can affect the sensitivity of MRD.^44^ Ultimately, in this study, we were able to confirm the correlation of sIL‐2R levels with tumour burden, to prove its value as a prognostic factor and as a tool for disease monitoring, with the potential to be tested in future works, ideally in a prospective fashion.

## AUTHOR CONTRIBUTIONS

FA collected data, wrote the manuscript, performed statistical analyses and created figures and tables; AC and CAC provided intellectual inputs, reviewed the manuscript and visited patients; SZ and LF collected data; ND, AS, LB, FF, MP, AT and AB provided intellectual inputs and reviewed the manuscript; LT and AV ideated the work, provided intellectual inputs and reviewed the manuscript. All authors approved the final manuscript for publication.

## FUNDING INFORMATION

This study was supported by Associazione Italiana per la Ricerca sul Cancro (A.I.R.C.) IG‐25024 to LT, Progetti di Rilevanza Nazionale PRIN PNRR (P2022PSMX4) to AV, Ricerca per Credere nella Vita (RCV) odv, Padua Italy.

## CONFLICT OF INTEREST STATEMENT

FA, AC, ND, SZ, CAC, LF, LB, FF; MP, AB, PF, AV and LT have no conflicts of interest to declare within this study.

## ETHICS STATEMENT

The study was approved by the local ethics committee (number: 4218/A0/17) and was conducted in accordance with the declaration of Helsinki.

## PATIENTS CONSENT STATEMENT

All patients provided written informed consent.

## Supporting information


Data S1.

## Data Availability

The datasets generated and analysed for the study are not publicly available due to data protection policies. Access to data is generally strictly limited to the researchers who have obtained permission for data processing; for further information, please contact the corresponding author.
