# Supplementary material for: Serum soluble interleukin‐2 receptor levels in hairy cell leukaemia as a marker of tumour burden with prognostic value and as a tool for disease monitoring
Source: Br J Haematol. 2025 Jul 29;207(4):1357–65. doi: 10.1111/bjh.70059 (PMC12512071; doi:10.1111/bjh.70059)

**Supplementary Table 1:** Baseline patients’ demographic and biological characteristics.

| **Median age, years (range; IQR)** | 59.6 (33.5 – 88.2; 20.4) |
| --- | --- |
| **Sex (%)** |  |
| M | 61 (82.4%) |
| F | 13 (17.6%) |
| **WBC x 10^9^/L (range; IQR)** | 3.08 (0.64 – 10.2; 2.19) |
| **Circulating hairy cells** **x 10^9^/L** **(range; IQR)** | 0.36 (0.002 – 5.4; 0.66) |
| **Circulating hairy cells % (range; IQR)** | 10.6 (0.1 – 64.5; 15) |
| **Bone marrow infiltration % (range; IQR)** | 70 (2 – 100; 35) |
| **LDH, U/L (range; IQR)** | 190 (63 – 711; 102.5) |
| **Beta-2 microglobulin, mg/L (range; IQR)** | 2.1 (1.32 – 4.25; 0.88) |
| **Splenomegaly (%)** |  |
| Present | 38 (51.2) |
| Absent | 34 (45.9) |
| Not available | 2 (2.7) |
| **Spleen diameter, cm (range; IQR)** | 14 (9 – 26; 4.88) |
| **Splenomegaly evaluation method (%)** |  |
| Ultrasonography | 51 (68.9) |
| CT scan | 14 (18.9) |
| Physical examination | 7 (9.5) |
| **CD38 expression** |  |
| Positive | 22 (29.7) |
| Negative | 40 (54.1) |
| Not available | 12 (16.2) |

WBC: white blood-cell count; LDH: lactate dehydrogenase; IQR: interquartile range.

**Supplementary Table 2:** Results of univariate logistic regression for response depth (CR vs. PR/SD)

|  | **OR** | **95% CI** | **P-value** |
| --- | --- | --- | --- |
| Post-therapy log(sIL-2R) | 0.079 | 0.013 – 0.28 | **<0.001** |
| Pre-therapy log(sIL-2R) | 0.64 | 0.21 – 1.65 | 0.39 |
| Age | 1.06 | 0.99 – 1.15 | 0.09 |
| CD38+ | 1.67 | 0.32 – 12.5 | 0.57 |
| WBC count | 0.91 | 0.65 – 1.32 | 0.57 |
| Splenomegaly | 0.42 | 0.08 – 1.90 | 0.27 |
| Spleen diameter | 0.83 | 0.65 – 1.04 | 0.11 |
| LDH | 1.00 | 0.99 – 1.02 | 0.44 |
| B2M | 8.2 | 0.68 – 487.44 | 0.19 |
| % of circulating HCs before therapy | 0.95 | 0.88 – 0.99 | **0.02** |
| % of bone marrow HCs before therapy | 0.99 | 0.95 – 1.03 | 0.62 |
| Disease status (ND vs. R/R) | 1.10 | 0.05 – 7.90 | 0.94 |

OR: odds ratio; WBC: white blood cell count; LDH: lactate dehydrogenase; B2M: beta-2 microglobulin; HCs: hairy cells; ND: newly diagnosed; R/R: relapsed/refractory; CR: complete remission; PR: partial remission; SD: stable disease; statistically significant p-values indicated in bold

**Supplementary Table 3:** Results of multivariate logistic regression for response depth (CR vs. PR/SD)

|  | **OR** | **95% CI** | **P-value** |
| --- | --- | --- | --- |
| Post-therapy log(sIL-2R) | 0.10 | 0.016 – 0.36 | **0.003** |
| % of circulating HCs before therapy | 0.99 | 0.94 – 1.04 | 0.81 |

HCs: hairy cells; CR: complete remission; PR: partial remission; SD: stable disease; statistically significant p-values indicated in bold

**Supplementary Table 4:** Univariate analysis for OS

|  | **HR** | **95% CI** | **P-value** |
| --- | --- | --- | --- |
| Post-therapy log(sIL-2R) | 1.34 | 0.37 – 4.92 | 0.66 |
| Pre-therapy log(sIL-2R) | 2.76 | 0.69 – 11.0 | 0.15 |
| Age | 1.13 | 1.03 – 1.26 | **0.012** |
| CD38+ | 2.67 | 0.44 – 16.1 | 0.29 |
| WBC count | 1.08 | 0.73 – 1.61 | 0.69 |
| Splenomegaly | 4.41 | 0.49 – 39.6 | 0.19 |
| Spleen diameter | 1.03 | 0.81 – 1.32 | 0.79 |
| LDH | 0.99 | 0.97 – 1.01 | 0.16 |
| B2M | 8.92 | 0.60 – 133.6 | 0.11 |
| % of bone marrow HCs before therapy | 1.01 | 0.96 – 1.06 | 0.68 |
| MRD + | NA | NA | NA |
| Disease status (ND vs. R/R) | 0.37 | 0.06 – 2.12 | 0.28 |
| Response depth (CR vs PR) | NA | NA | NA |

HR: hazard ratio; WBC: white blood cell count; LDH: lactate dehydrogenase; B2M: beta-2 microglobulin; HCs: hairy cells; ND: newly diagnosed; R/R: relapsed/refractory; CR: complete remission; PR: partial remission; NA: not applicable due to low events; statistically significant p-values indicated in bold

**Supplementary Table 5:** Values of sensitivity, specificity, PPV, NPV and relative Youden’s index for the 730 kU/L (ULN) cutoff at different time points

|  | **730 kU/L cutoff (ULN)** | | | | |
| --- | --- | --- | --- | --- | --- |
| **Time in years** | **Sensitivity** | **Specificity** | **PPV** | **NPV** | **Youden’s Index** |
| t = 1 | 100% | 65.5% | 4.68% | 100% | 0.66 |
| t = 2 | 100% | 66.0% | 14.24% | 100% | 0.66 |
| t = 3 | 100% | 65.9% | 18.64% | 100% | 0.66 |
| t = 4 | 84% | 64.9% | 27.81% | 96.28% | 0.49 |
| t = 5 | 75% | 66.7% | 34.77% | 91.73% | 0.42 |
| t = 6 | 78% | 61.9% | 37.10% | 90.84% | 0.40 |
| t = 7 | 82% | 68.8% | 48.71% | 91.23% | 0.51 |
| t = 8 | 85% | 63.6% | 51.71% | 89.98% | 0.49 |
| t = 9 | 85% | 70.0% | 56.48% | 90.81% | 0.55 |
| t = 10 | 70% | 71.4% | 60.23% | 79.01% | 0.41 |

PPV: positive predictive value; NPV: negative predictive value

**Supplementary Table 6:** sIl-2R cutoff values with the best Youden index for each time point analyzed in the time dependent ROC curve analysis

| **Time in years** | **sIL-2R value (kU/L)** | **Sensitivity** | **Specificity** | **Youden Index** |
| --- | --- | --- | --- | --- |
| t = 1 | 4275 | 100% | 100% | 1 |
| t = 2 | 2628 | 100% | 100% | 1 |
| t = 3 | 842 | 100% | 82.98% | 0.83 |
| t = 4 | 842 | 84.39% | 83.78% | 0.68 |
| t = 5 | 1139 | 64.89% | 96.67% | 0.61 |
| t = 6 | 827 | 78.26% | 85.71% | 0.64 |
| t = 7 | 827 | 81.79% | 87.50% | 0.69 |
| t = 8 | 827 | 84.61% | 90.91% | 0.75 |
| t = 9 | 827 | 84.61% | 90% | 0.74 |
| t = 10 | 476 | 92.98% | 57.14% | 0.57 |

**Supplementary Figure 1**: Boxplot showing the difference between median pre- and post-therapy sIL-2R levels in patients treated with purine analogues.


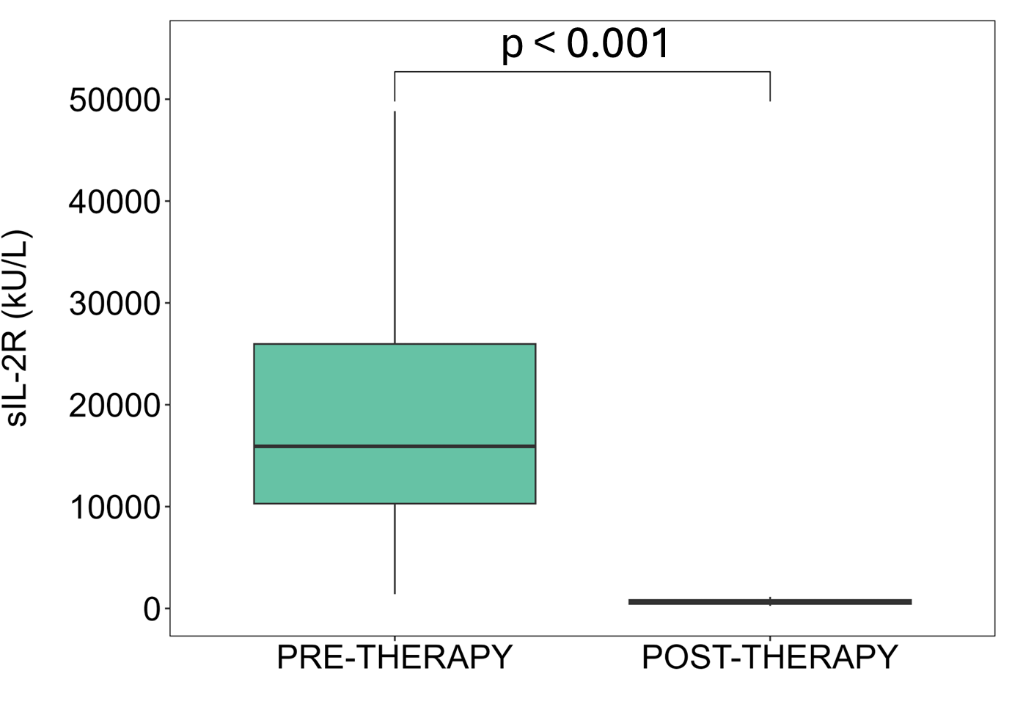


**Supplementary Figure 2**: Boxplot showing the difference between median pre- and post-therapy sIL-2R levels in patients treated with R-Vemurafenib.


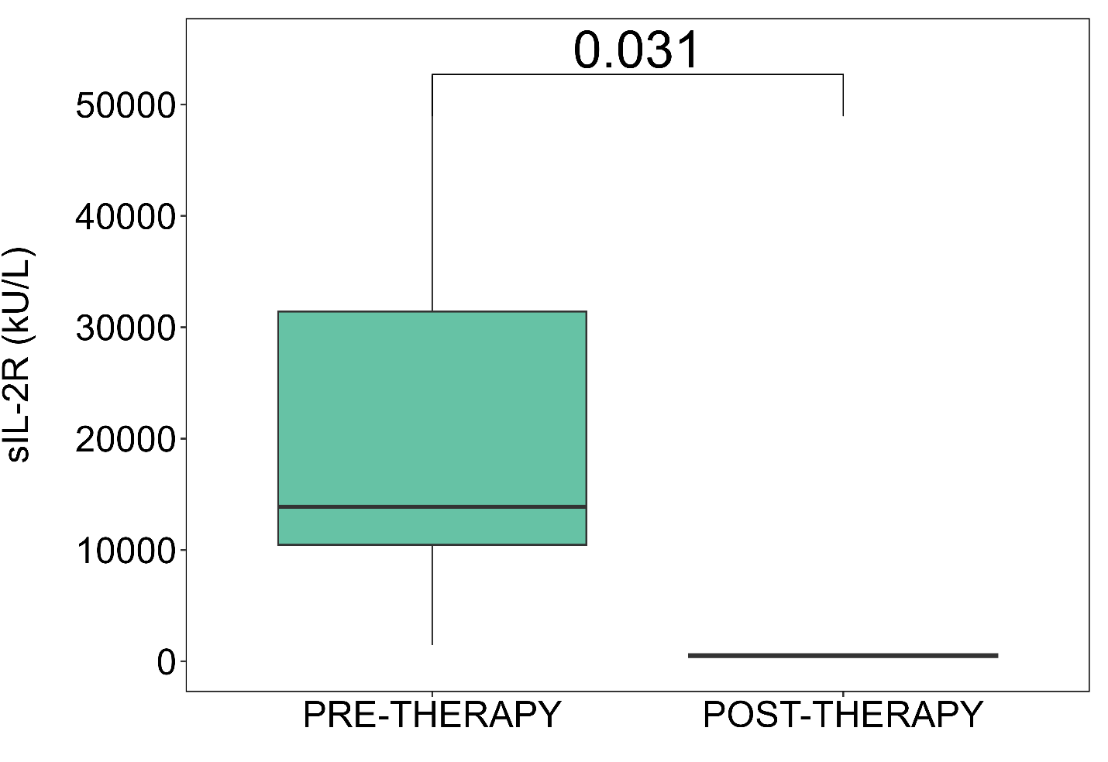


**Supplementary figure 3**: Scatterplot showing results of the maximally selected rank statistics analysis


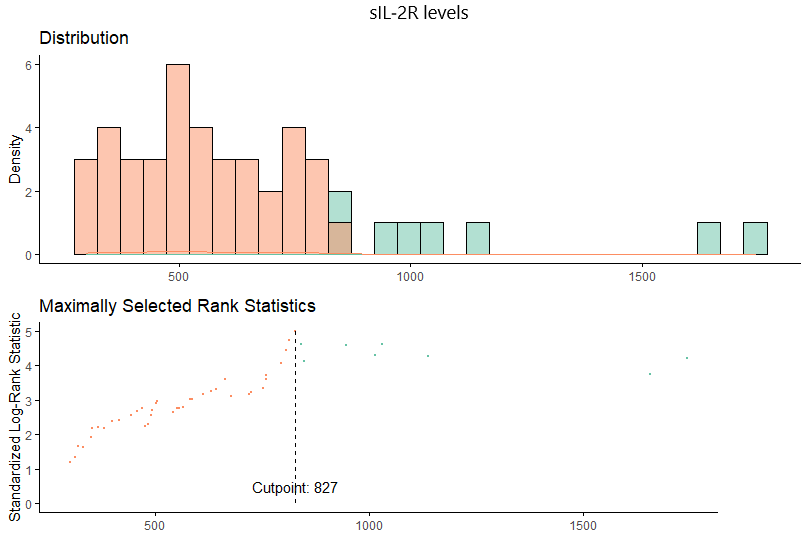


**Supplementary figure 4**: Survival curves according to the 827 kU/L cutoff in newly diagnosed patients


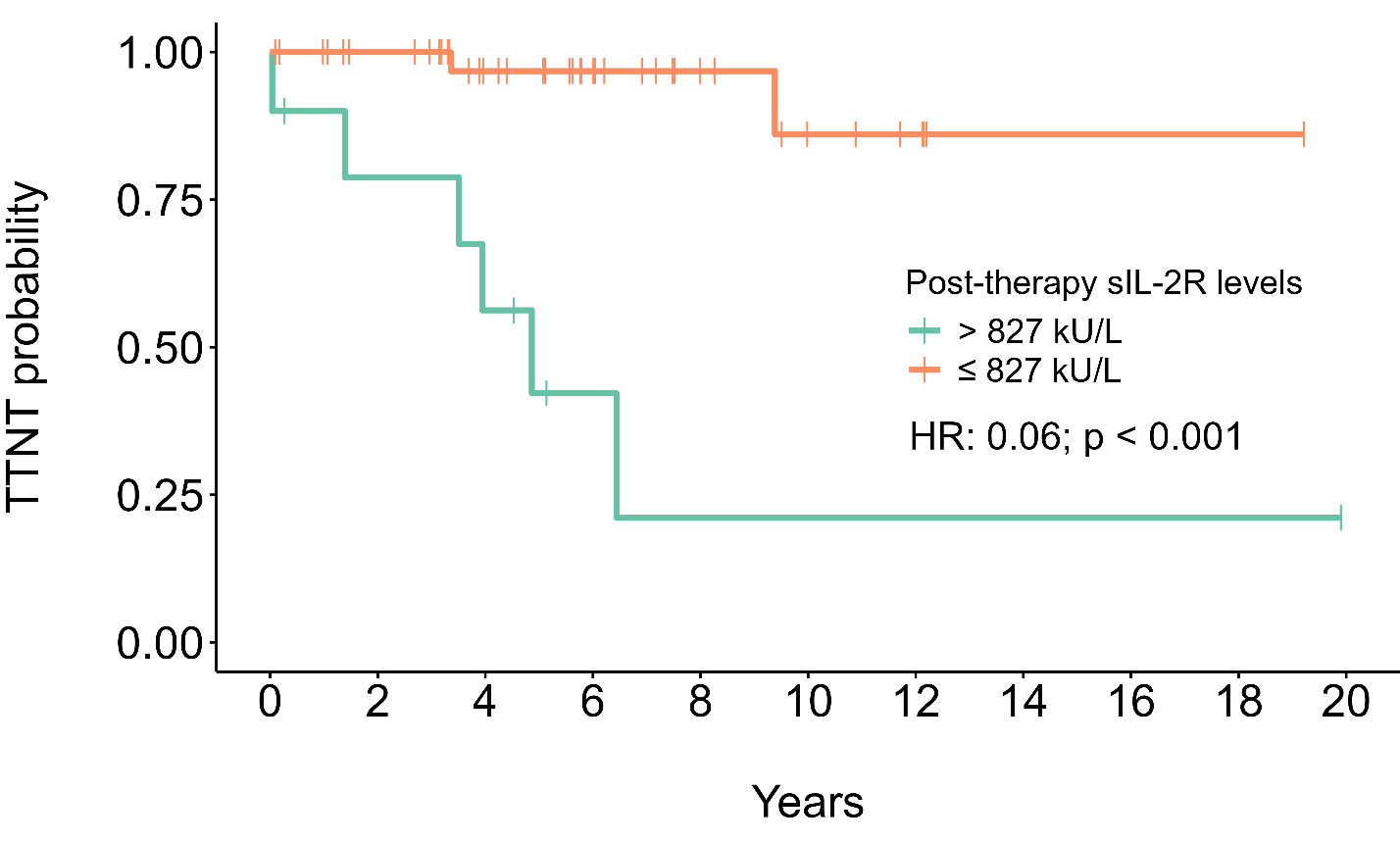


**Supplementary figure 5**: Survival curves for patients achieving post-therapy sIL-2R levels > 730 kU/L vs. ≤ 730 kU/L


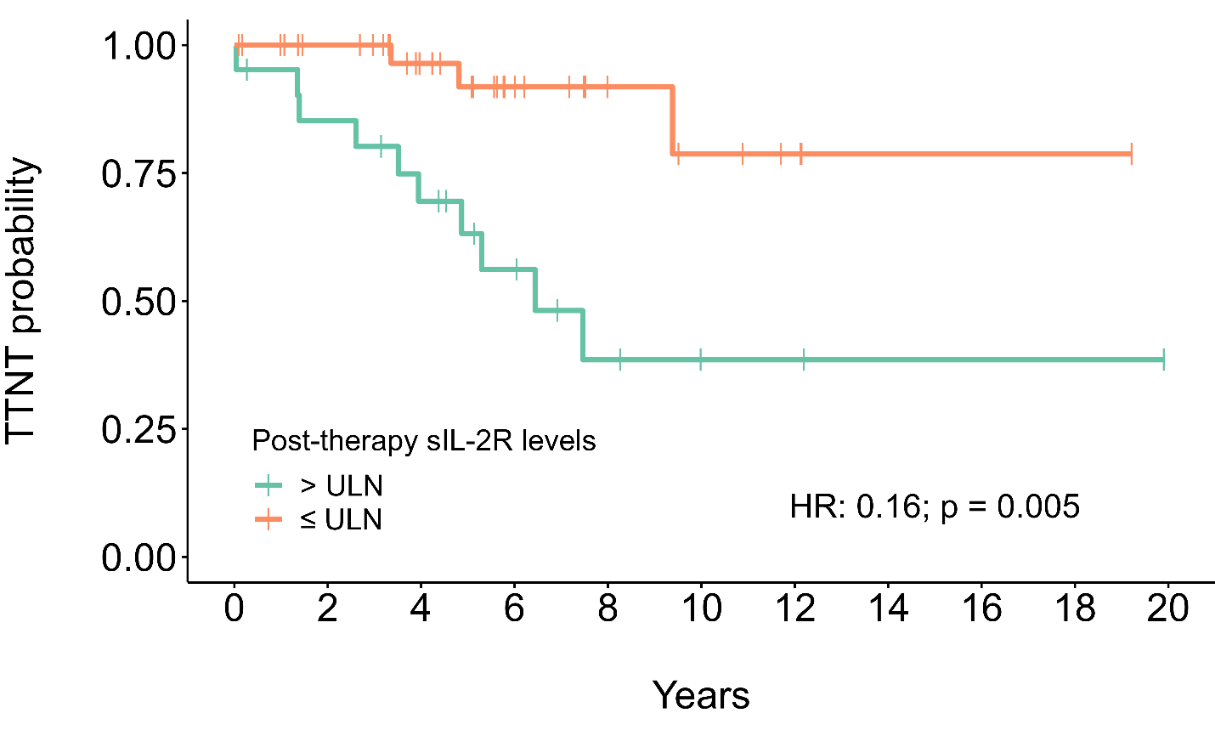


ULN: Upper limit of normal (730 kU/L)

**Supplementary figure 6**: Survival curves based on the reduction between pre- and post-therapy sIL-2R levels with a cutoff of ≥ 3-log reduction


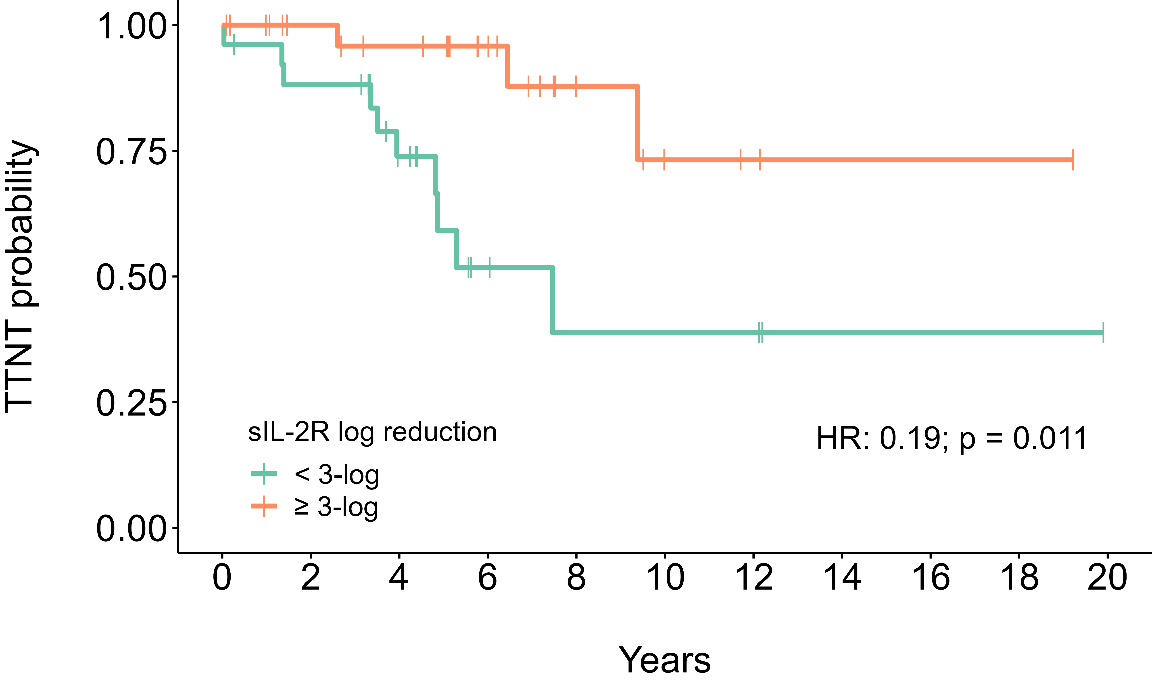

Supplement: Supplementary file 1 — Data S1. [file BJH-207-1357-s001.docx]
